# Physicians’ working conditions in hospitals from the students’ perspective (iCEPT-Study)—results of a web-based survey

**DOI:** 10.1186/s12995-016-0094-9

**Published:** 2016-02-19

**Authors:** Jan Bauer, David A. Groneberg

**Affiliations:** Institute of Occupational, Social and Environmental Medicine, Goethe-University Frankfurt, Theodor-Stern-Kai 7, 60329 Frankfurt am Main, Germany

**Keywords:** Student, Physician, Hospital, Distress, Satisfaction

## Abstract

**Background:**

Medical students undergo numerous clinical clerkships. On these occasions they are confronted with current working conditions in hospitals. Because of the many implications of the students’ perceptions of these working conditions, it is important to assess those. Hereby the focus was put on the students’ perception of their supervising physician.

**Methods:**

This study is a part of a prospective anonymized web-based survey (iCEPT-Study). The study was conducted in Germany among medical students after their clinical rotations. 1587 medical students took part in this study (63,0 % female and 37,0 % male). 11259 were invited to take part (response rate of 14,1 %). In this study a questionnaire was used which was based on the Effort-Reward-Imbalance (ERI) model and the Job-Demand-Control (JDC) model. A mathematical calculated ratio (ER- and JDC-Ratio; combined as ‘ER/JDC-Ratio’) was used to measure the students’ perceptions of working conditions, namely distress (primary outcome). As a secondary outcome perceived job satisfaction was measured.

**Results:**

Distress was perceived by 67.4 % (95 %-CI: 65.1|69.7) of the students. 54.1 % (95 %-CI: 51.7|56.6) of polled students stated that their supervising physician seemed to be very satisfied with his job. Analysis of age distribution revealed that the proportion of students’ who perceived their supervising physician as very satisfied with his job dropped from 72.5 % among under 20-year olds to 63.0 % among 20–24-year olds and was at 44.5 % among the over 30-year olds. Looking at the specialty, the specialty of surgery was rated with the highest distress prevalence (ER/JDC-Ratio > 1): 81.3 % of students stated that their supervising surgeon encountered unfavorable working conditions.

**Conclusion:**

Two out of three medical students rated the physicians working conditions as stressful. This implicates that already in this early phase of their career the majority of medical students get to know the hospital as an unfavorable workplace concerning working conditions. To facilitate the transition from medical schools to hospitals working conditions of physicians must be improved.

## Background

There are three important aspects examining physicians’ working conditions in the context of medical students: First, the way current working conditions are exemplified to medical students through the physicians. Second, the corresponding students’ perception of these working conditions. Third, thereby arising expectations of medical students regarding their future working conditions. The first and latter aspects has already been examined in many studies [1–5]. However research about the second aspect is rare, which is why the study focus has been put on students’ perception of physicians’ working conditions.

Regarding expectations on working conditions, a survey among German medical students in 2010 [4] showed that the work-life-balance is of outstanding importance: 96 % of respondents stated that combining family and work is important. Furthermore, 60.9 % of respondents would like to work part-time (women: 77.2 %; men: 32.1 %). Regarding the workplace, 77.7 % of respondents attested the hospital to be an attractive workplace. These trends have been confirmed in several studies [2, 3, 5–7].

In 1996 Bland et al. developed a theoretical model [8] trying to explain the specialty choice of medical students: the so called ‘Bland-Meurer model’. Therein the author distinguished two major reasons for a certain specialty choice: On the one hand the ‘needs to satisfy’ and on the other hand the ‘perception of specialty’. Factors playing a role in the perceptions of specialty are workload, patient contact and job satisfaction of the observed physicians.

Taken as a whole, the perception of working conditions (and therefore the specialty choice) is influenced by the direct observation of physicians on the one hand and by information during medical education obtained by fellow students, media or physicians on the other [8–11].

The physicians themselves seemed to show high levels of distress, as shown in several studies [12–14]. However, whether the respective students’ perception matches the physicians’ self-perception cannot be judged with current literature. Considering the implication the students’ perceptions of working conditions have on the specialty choice, a comparative evaluation is of great significance, especially in times of a shortage of qualified physicians. This is the case for example in Germany: The ’deutsche Krankenhausinstitut’ [15] predicted further personnel requirements of 37370 physicians until the year 2019. The ’WifOR Institut’ in cooporation with PricewaterhouseCoopers [16] forecasted further personnel requirements of 56000 physicians until the year 2020.

Since the polled students of the iCEPT-Study are from Germany some short facts will be presented about German medical students: In Germany there were 82289 medical students in the winter semester of 2011/12 [17]. The number of annual graduates sunk from 11987 to 8659 during the years 1994 to 2006. In the year 2010 there were 9844 graduates [18]. Of the graduates 92 % work as physicians one year after their final exam, according to the ‘Medizinerreport 2012’ of HIS GmbH [19]. After 10 years only 86 % would work as a physician. Furthermore the official success rate of German medical students from the year 2000 to 2009, meaning the rate of students who graduated successfully, was 95 % [20].

The in the beginning mentioned second aspect, the students’ perception of the physicians’ working conditions, hasn’t been subject of an investigation so far and therefore chosen as study focus.

## Methods

This study was part of the iCept-Study (iCept: Neologism of ‘i percept’). The respective complete study protocol has already been published [21]. Ethical approval has been obtained.

The iCept-Study used two stress models as the theoretical substructure: The Effort-Reward-Imbalance (ERI) model [22] and the Job-Demand-Control (JDC) model [23, 24]. Both models introduce two parameters, which in case of an imbalance (Ratio > 1) of one parameter (‘effort’ in the ERI model and ‘job-demand’ in the JDC model) lead to unfavorable working conditions and therefore distress (defined as negative, chronic stress with negative impact on health) [25]. Therefore distress is present in case of an Effort-Reward (ER)—Ratio > 1 and/or a Job-Demand-Control (JDC)—Ratio > 1. Here both stress models were combined and referred to with the term ‘ER/JDC-Ratio’ defined by an ER-Ratio > 1 and/or a JDC-Ratio > 1 since thereby a valid decision about the presence of distress can be made.

### The iCept-Questionnaire

The iCept-Study has been conceived as an online survey. The items of the iCept-Questionnaire were taken from two established and validated questionnaires: On the one hand the ‘Kurz-Fragebogen zur Arbeitsanalyse’ (KFZA) of Prümper et al [26] and on the other hand the ERI-Questionnaire of Siegrist et al [27]. Both questionnaires have often been used in hospitals [28–30]. The overall job satisfaction was measured by a single item (JS1) taken from the ’Job Diagnostic Survey’ (JDS) of Schmidt et al [31]. A meta-analysis showed that a single-item measure was as reliable and convincing as a scale measure with a correlation of r = 0,67 [32].

Since in this study students were asked to rate the working conditions of physicians, the items had to be adapted to the changed perspective: From first person singular to third person singular. Thus there are only grammatical differences, without changes to content.

### Mathematical evaluation

The items of the iCept-Questionnaire were summed up into scales according to the stress models of Siegrist and Karasek. In addition scale values were calculated and thereof a ratio was built. The scale values can vary depending on their respective number of items:

- Scale value ‘effort’ (x_eff_): 4 ≤ x_eff_ ≤ 16
- Scale value ‘job demand’ (x_job_): 4 ≤ x_job_ ≤ 16
- Scale value ‘reward’ (x_rew_): 5 ≤ x_rew_ ≤ 20
- Scale value ‘control’ (x_con_): 3 ≤ x_con_ ≤ 12

Because of the varying number of items, corrections factors were introduced: c_eri_ = 1.25 (5/4) for the scale ‘effort’ and c_jdc_ = 0.75 (3/4) for the scale ‘job-demands’.

$$ ER- Ratio=\frac{x_{eff}}{x_{rew}} \times {c}_{eri}\kern1em JDC- Ratio=\frac{x_{job}}{x_{con}} \times {c}_{jdc} $$

### Statistical data analysis

The statistical analysis has been performed using SPSS Version 21. The following tests have been used to test for significant differences: Mann-Whitney-U-Test for two measurement series, Kruskal-Wallis-Test for more than two measurement series and the Chi-Quadrat-Test for categorical criteria. Furthermore the odds ratio respectively the arithmetic average difference has been calculated with the t-test including the 95 %-confidence interval. With these parameters a conclusion could be drawn about strength and direction of differences.

### Study participants

For the purposes of the iCept-Study a total number of 11259 medical students in Germany were invited via e-mail to take part in this study. In total 1587 students participated. This corresponds to a response rate of 14.1 % or in relation to all 82289 medical students in Germany (basic sample) to 1.9 %. The Federal Statistical Office of Germany obtains the data used to compare the iCept-sample with the basic sample [17].

**Fig. 1:**
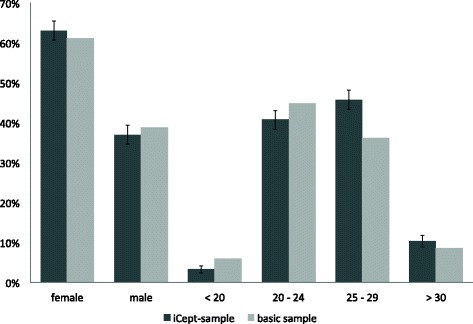
Comparison of major criteria of ICept-sample and basic sample

In Fig. 1 a comparison of the iCept-sample and the basic sample is given. The average age of the iCept-sample was 25.3 years (SD: 3.6 years), In the basic sample the average age was 25.2 years. Regarding the study phase, 12.1 % of respondents were in their first or second year (1./2. year; i.e. ‘preclinical’), 49.7 % of respondents were in the third, fourth or fifth year (3./4./5. year; i.e. ‘clinical’) and 38.2 % in their final year (6. year; i.e. ‘elective year’).

Since the students were asked to rate the physicians’ working conditions during their internship, the following data analysis do not reflect the students’ working conditions but the physicians’ working conditions in the perception of medical students.

## Results

The analysis of data showed that for 54.1 % (95 %-CI: 51.7|56.6) of polled students their supervising physician seemed to be very satisfied with his job. Unfavorable working conditions in form of an ER/JDC-Ratio > 1 (distress) perceived 67.4 % (95 %-CI: 65.1|69.7) of the students. Furthermore, 41.7 % (95 %-CI: 38.1|44.7) of polled students stated, that their supervising physician seemed to be very satisfied despite distress. In Table 1 an overview of the results is displayed.

**Table 1:** Overview on results, according to gender, study phase and age; *p < 0.05 **p < 0.01 ***p < 0.001

	Total	Gender		Study phase (year)			Age			
		*male*	*female*	*1./2.*	*3./4./5.*	*6.*	*<20*	*20 - 24*	*25 - 30*	*>30*
	n = 1.587	n = 587	n = 1.000	n = 192	n = 788	n = 607	n = 51	n = 646	n = 726	n = 164
	n (%)	n (%)	n (%)	n (%)	n (%)	n (%)	n (%)	n (%)	n (%)	n (%)
		OR (95 %-CI)	OR (95 %-CI)	OR (95 %-CI)	OR (95 %-CI)	OR (95 %-CI)	OR (95 %-CI)	OR (95 %-CI)	OR (95 %-CI)	OR (95 %-CI)
*ER- and JDC-Ratio > 1*	1.069 (67,4)	381 (64,9)	688 (68,8)	123 (64,1)	517 (65,6)	429 (70,7)	32 (62,7)	420 (65,0)	509 (70,1)	108 (65,9)
		1	1,19 (0,96|1,48)	1	1,07 (0,77|1,49)	1,35 (0,96|1,91)	1	1,10 (0,60|1,99)	1,39 (0,77|2,51)	1,15 (0,60|2,20)
		-	-	-	1	1,26 (1,01|1,59)*	-	1	1,26 (1,01|1,58)*	1,04 (0,72|1,49)
		-	-	-	-	-	-	-	1,22 (0,85|1,74)	1
*JS1: "very satisfied"*	859 (54,1)	339 (57,8)	520 (52,0)	129 (67,2)	461 (58,5)	269 (44,3)	37 (72,5)	407 (63,0)	342 (47,1)	73 (44,5)
		1,26 (1,03|1,55)*	1	2,57 (1,83|3,62)***	1,77 (1,43|2,19)***	1	3,30 (1,66|6,55)***	2,12 (1,50|3,00)***	1,11 (0,79|1,56)	1
		-	-	1,45 (1,04|2,03)*	1	-	2,97 (1,58|5,58)***	1,91 (1,54|2,37)***	1	-
		-	-	-	-	-	1,55 (0,82|2,93)	1	-	-

The data were analyzed by the following students’ characteristics: Gender, age, study phase and specialty.

### Gender

There were no significant gender specific differences regarding distress (p = 0,110): 68.8 % of female and 64.9 % of male students rated the physicians working conditions as stressful (ER/JDC-Ratio > 1). Solely the aspect of job satisfaction revealed significant (*p* < 0.05) differences: For 52.0 % of female students and 57.8 % of male students their supervising physician seemed to be very satisfied with his job. This corresponded with an odds ratio of 1.26 (95 %-CI: 1.03|1.55).

### Age

Taking the students’ age in the focus the data analysis showed a correlation between job satisfaction and the four generated age groups: The proportion of students’ who perceived their supervising physician as very satisfied with his job dropped from 72.5 % among under 20-year olds to 63.0 % among 20–24-year olds, to 47,1 % among 25–30-year olds and was at 44.5 % among the over 30-year olds. Therefore the odds ratio of under 20-year olds to over 30-year olds was 3.30 (95 %-CI: 1.66|6.55). Regarding distress prevalence only the 25–30-year olds had a significant higher distress compared to the 20–24-year olds (65.0 % to 70.1 %). This corresponded with an odds ratio of 1.26 (95 %-CI: 1.01|1.58; *p* < 0.05).

### Study phase

The analysis of study phases revealed that for first- and second-year students their supervising physician seemed more often satisfied with his job than for students in higher study phases: This statement applied to 67.2 % of first-and second-year students, 58.5 % of third-, fourth- and fifth-year students and 44.3 % of final-year students. The odds ratio of third-, fourth- and fifth-year students to final-year students was 2.57 (95 %-CI: 1.83|3.62; *p* < 0.001). Concerning distress prevalence, the third-, fourth- and fifth-year students perceived their supervising physician less often stressed out (ER/JDC-Ratio > 1) with a prevalence of 65.6 % compared to 70,7 % of final-year students who stated this. With an odds ratio of 1.26 (95 %-CI: 1.01|1.59; *p* < 0.05) this finding was significant.

### Specialty

**Fig. 2:**
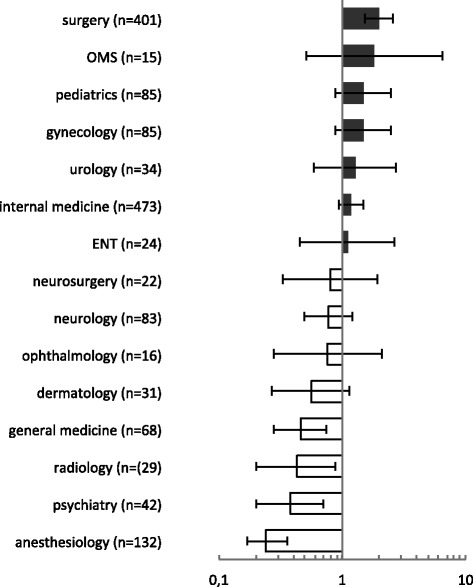
Odds ratio of distress prevalence compared to the average, according to specialty; ENT (ear-nose-throat), OMS (oral and maxillofacial surgery)

This chapter focuses on working conditions in different specialties from the students’ perspective. The specialty of surgery was rated with the highest distress prevalence (ER/JDC-Ratio > 1): 81.3 % of students stated that their supervising surgeon encountered unfavorable working conditions. Compared to the average of 64.4 % this corresponded with an odds ratio of 1.99 (95 %-CI: 1.51|2.61; *p* < 0.001). The lowest distress prevalence in the perception of students was present in the specialty of anesthesiology with 34.8 % and a corresponding odds ratio to the average of 0.24 (95 %-CI: 0.17|0.36; *p* < 0.001). Also a significant lower prevalence was present in the specialty of psychiatry (45.2 %) and radiology (48.3 %). More details and specialties are displayed in Fig. 2. As this figure indicates, there were substantial differences between specialties compared to the average and even more if compared directly: The odds ratio of anesthesiology to surgery regarding distress was 0.12 (95 %-CI: 0.08|0.19).

**Fig. 3:**
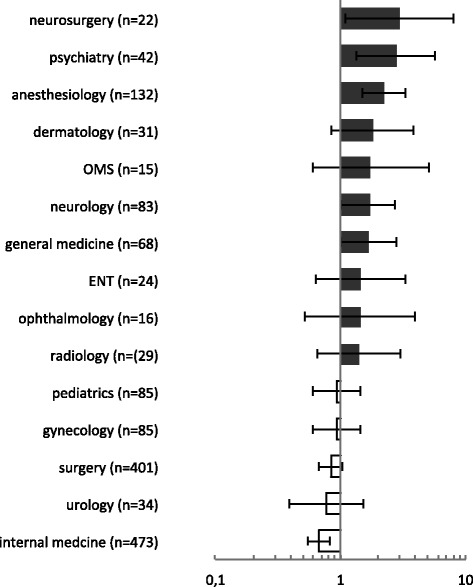
Odds ratio of job satisfaction prevalence compared to the average, according to specialty; ENT (ear-nose-throat), OMS (oral and maxillofacial surgery)

The further analysis of specialties regarding job satisfaction revealed similar differences: From the students’ perspective the neurosurgeons seemed to be significantly more often satisfied with their job. 77.3 % of students stated that their supervising neurosurgeon was very satisfied with his job. Compared to the average of 54.1 % this corresponded with an odds ratio of 2.94 (95 %-CI: 1.08|8.01; *p* < 0.05). In the specialty of anesthesiology 72.0 % of students stated this with an odds ratio to the average of 2.22 (95 %-CI: 1.50|3.29; *p* < 0.001). Physicians in the specialty of internal medicine seemed to be less often satisfied: 43.8 % of students stated that their supervising physician was very satisfied with his job. Again compared to the average this corresponded with an odds ratio of 0.67 (95 %-CI: 0.55|0.83; *p* < 0.001). If compared to neurosurgery this corresponded with an odds ratio of 0.23 (95 %-CI: 0.08|0.63; *p* < 0,001). More specialties are displayed and compared to the average in Fig. 3.

## Discussion

Whether the iCEPT-data can be seen as representative will be discussed first: Considering the response rate of 14.1 %, a selection-bias due to non-responder is possible. The response rate is relatively low compared to other web-based studies [33], raising the question of reliability. However the absolute number of 1587 participants is relatively high. Although there is a relatively high absolute number of participating students (1587) the representativeness of the data must be considered as critical. However, there are high congruencies of the iCEPT-sample in major characteristics with the basic sample, which could be seen as an indicator of representativeness.

As in the introduction indicated, there is a lack of sufficient international and national data regarding students’ perception of working conditions (according to gender, age or study phase) to compare these results with. Discussing the data according to specialty, the data will be compared to the specialty registration of physicians from the year 2013 in Germany [34]. This will be taken as an indirect indicator of students’ perception of working conditions. In the iCEPT-Study the specialty of anesthesiology had relatively high job satisfaction prevalence and at the same time relatively low distress prevalence. The proportion of anesthesiology registration compared to all specialty registrations was 8.5 % and therefore anesthesiology came third. This seems to confirm the results of the iCEPT-study. On the other hand, first in specialty registration was internal medicine and second surgery. Both had relatively high distress prevalences and low job satisfaction prevalences in the iCEPT-Study. This seems to call the data into question. But there are multiple factors limiting this comparison: First the time lag of the final study exam to the specialty registration (at least 4–6 years). Second, there are many factors to be considered when making a specialty choice, the students’ perception of working conditions only being one of them [8]. Still the perception of working conditions plays an important role: In a study from the year 2011 which focused on specialty choice of medical students, working conditions were the number two reason for students to choose their specialty [35]. However, the focus of our study was the perception of working conditions and not specialty choice, therefore despite similarities, the comparison of both studies has limitations.

The outlined results of students’ perspective on physicians’ working conditions will now be compared to the physicians’ view on their own working conditions. For this purpose data of 7090 physicians from another published part of the iCEPT-study will be taken as the comparative data [14, 36–38]. Therefore the reference value of job satisfaction and distress among physicians is as followed: 53.9 % of polled physicians encountered distress (ER/JDC-Ratio > 1) and 55.8 % were very satisfied with their job. For these data the comparison with the students’ data is indicated by the odds ratio: Among students there seem to be higher distress prevalences present than among physicians with an odds ratio of 1.76 (95 %-CI:1.57|1.98; *p* < 0,001). Especially female students seemed to rate the working conditions more often in form of an ER/JDC-Ratio > 1 (OR: 1.88; 95 %-CI:1.64|2.17; *p* < 0.001). Regarding the age, the 25-30-year old students stated a significant higher distress prevalence than physicians themselves (OR: 2.00; 95 %-CI:1.70|2.36; *p* < 0.001). The overall job satisfaction seemed to be perceived similarly among students and physicians, since there was no significant difference. Solely female students in contrast to male students seemed to perceive the physicians less often as satisfied with their job than the physicians themselves. The analysis of students’ age regarding perception of job satisfaction revealed that especially for the under 20-year old students their supervising physician seemed more often satisfied than the physicians stated themselves (OR: 2,09; 95 %-CI: 1,13|3,88; *p* < 0.001). Studies [39] have shown that there is a strong correlation between job stressors such as lack of leader support and low job satisfaction. The gender differences in the perception of working conditions could be explained by differing expectations of working conditions resulting in differing numbers regarding part-time: in 2013 30,4 % of all female physicians in Germany worked part-time, whereas 11,8 % of male physicians worked part-time [40]. Also during medical education gender issues arise which could influence the perception of working conditions [41]. In regard to differences among the age groups, the cumulative time spent in hospitals as well as personal experiences could affect the perception. However in the presented study no causal factors were investigated and therefore no conclusion can be drawn about causal factors.

In an Australian study comparing the perception of students and consultants in the field of emergency medicine there were also significant differences: 22.4 % of students and 50.0 % of consultants (*p* < 0.05) said that the workload would be too high. Furthermore 95.5 % of consultants and 64.9 % of students (*p* < 0.001) said that being an emergency physician would be a rewarding career [42].

## Conclusion

The data set provided is valid and objective, giving clear insight on the students’ perception of working conditions. So far this is among the first studies focusing explicit on external perception of working conditions. Two out of three medical students rated the physicians working conditions as stressful. This implicates that already in this early phase of their career the majority of medical students get to know the hospital as an unfavorable workplace concerning working conditions. In order to keep medical employees interested in the hospital, this has to be changed. However, the supervising physicians still seemed to be quite often very satisfied with their job. The discrepancy of satisfaction and working conditions illustrate the need for more research on this topic. Focusing on specialties, there are substantial differences regarding distress and job satisfaction. Taking the implications into account which go along with the perception of working conditions for the future career and/or specialty choice of medical students, it is crucial to improve the very same. Hence the displayed data can be used for creating balanced working conditions according to the respective stress models.

## Abbreviations

CI: confidence interval
ENT: ear-nose-throat
ER: effort reward
ERI: effort reward imbalance
JD: job demand
JDC: job demand control
KFZA: abbreviation for “Kurz-Fragebogen zur Arbeitsanalyse” (short questionnaire for work analysis)
M: mean
OMS: oral and maxillofacial surgery
OR: odds ratio
SD: standard deviation
